# Expression and the Peculiar Enzymatic Behavior of the *Trypanosoma cruzi* NTH1 DNA Glycosylase

**DOI:** 10.1371/journal.pone.0157270

**Published:** 2016-06-10

**Authors:** Fernando Ormeño, Camila Barrientos, Santiago Ramirez, Iván Ponce, Lucía Valenzuela, Sofía Sepúlveda, Mainá Bitar, Ulrike Kemmerling, Carlos Renato Machado, Gonzalo Cabrera, Norbel Galanti

**Affiliations:** 1 Programa de Biología Celular y Molecular, Instituto de Ciencias Biomédicas, Facultad de Medicina, Universidad de Chile, Santiago, Chile; 2 Departamento de Bioquímica e Imunologia, ICB, Universidade Federal de Minas Gerais, Belo Horizonte, MG, Brazil; 3 Programa de Anatomía y Biología del Desarrollo, Instituto de Ciencias Biomédicas, Facultad de Medicina, Universidad de Chile, Santiago, Chile; Instituto Butantan, Laboratório Especial de Toxinologia Aplicada, BRAZIL

## Abstract

*Trypanosoma cruzi*, the etiological agent of Chagas’ disease, presents three cellular forms (trypomastigotes, epimastigotes and amastigotes), all of which are submitted to oxidative species in its hosts. However, *T*. *cruzi* is able to resist oxidative stress suggesting a high efficiency of its DNA repair machinery.The Base Excision Repair (BER) pathway is one of the main DNA repair mechanisms in other eukaryotes and in *T*. *cruzi* as well. DNA glycosylases are enzymes involved in the recognition of oxidative DNA damage and in the removal of oxidized bases, constituting the first step of the BER pathway. Here, we describe the presence and activity of TcNTH1, a nuclear *T*. *cruzi* DNA glycosylase. Surprisingly, purified recombinant TcNTH1 does not remove the thymine glycol base, but catalyzes the cleavage of a probe showing an AP site. The same activity was found in epimastigote and trypomastigote homogenates suggesting that the BER pathway is not involved in thymine glycol DNA repair. TcNTH1 DNA-binding properties assayed *in silico* are in agreement with the absence of a thymine glycol removing function of that parasite enzyme. Over expression of TcNTH1 decrease parasite viability when transfected epimastigotes are submitted to a sustained production of H_2_O_2_.Therefore, TcNTH1 is the only known NTH1 orthologous unable to eliminate thymine glycol derivatives but that recognizes and cuts an AP site, most probably by a beta-elimination mechanism. We cannot discard that TcNTH1 presents DNA glycosylase activity on other DNA base lesions. Accordingly, a different DNA repair mechanism should be expected leading to eliminate thymine glycol from oxidized parasite DNA. Furthermore, TcNTH1 may play a role in the AP site recognition and processing.

## 1. Introduction

*Trypanosoma cruzi* (*T*. *cruzi*), a hemoflagellate protozoan parasite, is the etiological agent of Chagas’ disease, an endemic pathology in Latin America [1]. Twenty eight million people are at risk of exposure to infection with an estimated 6–7 million chronic cases in 21 endemic countries and 20.000 deaths per year. It is the parasitic disease with greater economic burden in America due to its long chronicity [2]. Recently, the appearance of *T*. *cruzi* infected vectors in the USA [3] and the identification of more than 300.000 people carrying the parasite in that country [4] together with the globalization of Chagas disease through immigration [5] have converted this infection in a worldwide problem [2,6,7]. There are no effective, specific and safe drug treatments for this chronic illness and new therapeutic targets should be urgently developed [8].

Chagas’ disease is transmitted by *T*. *cruzi* infected triatomine insects that upon feeding on mammal blood, deposit feces with metacyclic infective trypomastigotes. The parasite enters the mammal body and invades tissue histiocytes; there, it must survive in the acidic parasitophorous vacuoles where free radicals are generated [9–11]. Survivor parasites leave that hostile environment entering the host-cell cytoplasm where they differentiate to amastigotes that, in the chronic infection are also under oxidative stress by inflammatory cells or by cardiomyocyte mitochondrial dysfunction [12–14]. After undergoing many cycles of multiplication surviving amastigotes differentiate back into mobile trypomastigotes which escape into circulation making their way to target tissues. Blood trypomastigotes may be ingested by triatomines and transformed to replicative epimastigotes in the vector’s midgut. Epimastigotes are also submitted to oxidative species during hemoglobin catabolism; those that survive multiply and move to the insect hindgut where they differentiate into infective metacyclic trypomastigotes [11,15–17]. Thus, the parasite is able to resist oxidative stress at different stages of its life cycle, suggesting a high efficiency of its DNA repair machinery.

The Base Excision Repair (BER) pathway is one of the main DNA repair mechanisms in other eukaryotes and in *T*. *cruzi* as well [18–23]. DNA glycosylases are enzymes involved in the recognition of oxidative DNA damage and in the removal of oxidized bases, constituting the first step of the BER pathway [24–26]. To date 11 different human DNA glycosylases have been characterized [19] and six of them are related to oxidative DNA damage repair (OGG1, NTH1, NEIL1, NEIL2, NEIL3 and MYH) [25,27]. In the *T*. *cruzi* genome there are four sequences coding for DNA glycosylases related to repair of oxidized DNA bases: the homologs of human NTH1, OGG1, MYH, and NEIL3. NTH1 protein has been described as a bifunctional enzyme that recognizes and removes pyrimidine oxidized derivatives, and then catalyzes the rupture of the DNA strand through an AP lyase activity [28–30]. To date, no studies have been reported describing the presence and involvement of a NTH1 enzyme in *T*. *cruzi* DNA repair. In the *T*. *cruzi* genome, a sequence orthologous to this enzyme (TcNTH1) is present. This sequence was cloned in an *Escherichia coli* expression vector and the recombinant TcNTH1 was purified in denaturing and in native conditions. Mice were immunized with the denatured purified protein and the obtained antibody was used to identify the TcNTH1 enzyme in the three cellular forms of *T*. *cruzi*. A TcNTH1-GFP fusion protein was localized in the parasite nucleus. For activity assays native enzyme purified from bacteria and from recombinant epimastigotes were incubated with labeled oligonucleotide probes presenting an oxidized base (thymine glycol) or an apurinic/apyrimidinic site (AP site), as substrates. TcNTH1 enzyme does not remove the thymine glycol base, but catalyzes the cleavage of the probe showing an AP site. The same activity was found in epimastigotes and trypomastigotes homogenates suggesting that the BER pathway is not involved in thymine glycol DNA repair. The absence of a thymine glycol removing function was also suggested by *in silico* assays of TcNTH1 DNA- binding properties.

Over expression of TcNTH1 does not modify parasite viability when TcNTH1 transfected epimastigotes are exposed to H_2_O_2_ for 30 minutes. However, parasite survival is decreased when those parasites are submitted to a sustained production of H_2_O_2_.

Therefore, TcNTH1 is the only NTH1 orthologous unable to eliminate thymine glycol derivatives but that recognizes and cuts an AP site, most probably by a beta-elimination mechanism.

## 2. Material and Methods

All animal experiments were carried out in accordance with Chilean law and approved by the Animal Experimental Committee at the Faculty of Medicine, Universidad de Chile.

Work with *Trypanosoma cruzi* cell lines was performed with the approval of the Biosafety Committee, Faculty of Medicine, University of Chile and following national (Bioseguridad 1ra edición, 1994, Comisión Nacional de Investigación Científica y Tecnológica, CONICYT, Chile) and international (Manual de Bioseguridad en Laboratorios, OMS, Ginebra 2005) guidelines.

H9C2 (2–1) cardiomyocyte cell line was obtained from ATCC CRL-1446 (Rattus norvegicus)

### 2.1 Parasite culture

*T*. *cruzi* epimastigotes (Y strain) were cultivated at 28°C in LIT medium (Liver Infusion Tryptose: 5 g/L liver extract, 3.97 g/L NaCl; 0.395 g/L KCl;3.12 g/L HPO_4_Na_2_, 2 g/L glucose; [31] supplemented with 10% fetal bovine serum (FBS), 20 μg/ml hemin, 100 U/ml penicillin and 100 μg/ml streptomycin. Trypomastigotes and amastigotes parasite forms were obtained from infected H9C2 (2–1) cardiomyocyte cell cultures (ATCC CRL-1446, Rattus norvegicus rat) maintained in DMEM supplemented with 10% active FBS at 37°C in 5% CO_2_ as described for RAW cells [23].

### 2.2 Preparation of a TcNTH1 polyclonal antibody

The *Tcnth1* DNA coding sequence was amplified from genomic *T*. *cruzi* DNA by PCR using Platinum Taq High Fidelity polymerase (Invitrogen) and the primers forward (5’-CGGGATCCATGAAGAAGCATGCGTTC-3’) and reverse (5’-CCCAAGCTTTCACCGGGTATCGACAT-3’). Those primers present restriction sites for *Bam*HI and *Hind*III enzymes in the sense and in the antisense sequences, respectively. The restriction digestion created cohesive ends for oriented ligation into the plasmid expression vector pQE-80L (Qiagen Inc., Valencia, CA). Recombinant plasmid was transformed into competent *Escherichia coli* BL21 (DE3) pLys S. Synthesis of recombinant His-TcNTH1 protein was induced with 1 mM IPTG and its identity was confirmed by mass spectrometry (MALDI-TOF). The resulting fusion protein, carrying an N-terminal 6xHis-tag sequence, was purified under denaturing conditions in a Ni-NTA (Invitrogen) resin column, following the manufacturer’s recommendations and used to immunize *Mus musculus* females [32].

### 2.3 TcNTH1 identification in *T*. *cruzi* cellular forms

TcNTH1 DNA glycosylase was identified by western blot assays in epimastigotes, amastigotes and trypomastigotes protein extracts using the specific mouse anti-TcNTH1 polyclonal antibody (see above). Briefly, parasite protein extracts were separated by electrophoresis in 15% acrylamide gels. Afterwards, proteins were transferred to nitrocellulose membranes and incubated with 5% bovine serum albumin (BSA) in PBS 0.05% Tween-20 at 4°C overnight. Subsequently, membranes were incubated for 2 h with the anti-TcNTH1 antibody in a 1:2000 v/v dilution in 1%BSA in PBS 0.05% Tween-20 at room temperature and afterwards with a secondary goat anti-mouse antibody coupled to horseradish peroxidase (HRP, Jackson Immuno Research Laboratories, Inc). Results were visualized by chemiluminescence. Identification of α-tubulin with a specific mouse monoclonal antibody (Sigma, cat n° T-5168) in a 1:10.000 v/v dilution in 1%BSA in PBS 0.05% Tween-20 was used as load control.

### 2.4 TcNTH1 pTREX plasmid constructions

*Tcnth1* DNA coding sequences were amplified by PCR and inserted in pTREX and pTREX-GFP vectors [33]. Fusion proteins with an 8 histidine Tag in the N-terminal or with the green fluorescent protein Tag (GFP) in the C-terminal region respectively, were produced. The primers used for His-TcNTH1 generation (pTREX vector) were: sense 5’-GCTCTAGAATGCACCATCACCATCACCATCACCATATGAAGAAGCATGCGTTCAAGC-3’ (which incorporates a sequence for 8xHis-tag) and antisense 5’-GCTCTAGATCACCGGGTATCGACATCTTC-3’. Both primers present restriction sites for *Xba*I enzyme. On the other hand, the primers used for TcNTH1-GFP generation (pTREX-GFP vector) were: sense 5’-GCTCTAGAATGAAGAAGCATGCGTTCAAGC-3’ and antisense 5’-CCCAAGCTTCCGGGTATCGACATCTTCGAT-3’. These primers present restriction sites for *Xba*I in the sense and for *Hind*III in the antisense sequences. The correct insertion of *Tcnth1* DNA sequence in each plasmid was confirmed by PCR, enzymatic digestion and automatic DNA sequencing (data not shown).

### 2.5 Transfection and overexpression of His-TcNTH1 and TcNTH1-GFP in *T*. *cruzi* epimastigotes

Epimastigotes in the exponential phase of growth were electroporated with pTREX-his-*Tcnth1* or pTREX-*Tcnth1-gfp* constructs and with pTREX-empty and pTREX-*gfp* control vectors. Briefly, parasites were washed in sterile PBS and resuspended in electroporation buffer (120 mM KCl, 0.15 mM CaCl, 10 mM K_2_HPO_4_, 25 mM Hepes, 2 mM EDTA, 5 mM MgCl_2_, pH 7.6). Afterwards 4x10^7^ parasites were separately incubated with 50–100 μg of each plasmid. The electroporation was performed at 0.3 kV and 500 μF in two pulses separated by 30 seconds maintaining the parasites on ice. Transfected epimastigotes were immediately transferred to 20% FBS LIT medium and, after 24 h, 250 μg/ml of G418 antibiotic was added and the antibiotic concentration was increased to 500 μg/ml at 72 h.

### 2.6 TcNTH1 recombinant protein generation

Recombinant TcNTH1 proteins were generated in transformed pQE80L-*his-Tcnth1 E*. *coli* cells and in transfected pTREX-*his-Tcnth1 T*. *cruzi* epimastigotes as described above. Both recombinant proteins were purified from bacteria or from epimastigote protein homogenates by affinity chromatography using a HisPur Ni-NTA resin (Thermo Scientific) in native conditions, following the manufacturer’s recommendations.

### 2.7 DNA glycosylase activity assay

The bifunctional DNA glycosylase activity of the purified native recombinant TcNTH1 from *E*. *coli* and from *T*. *cruzi* epimastigotes as well as from epimastigote and trypomastigote homogenates was determined using a 32 mer synthetic DNA oligonucleotide containing a thymine glycol residue at position 18 (5’-CCGGTGCATGACACTGT(Tg)ACCTATCCTCAGCG-3’; where Tg indicates thymine glycol). This oligo was labeled at the 5’ end with [γ-^32^P]ATP (3x10^3^Ci/mmol, 20 μCi per 40 pmol of substrate) using the DNA 5’ End Labeling System kit (Promega) and then hybridized with unlabeled complementary oligonucleotide (5’-CGCTGAGGATAGGT(A/G)ACAGTGTCATGCACCGG-3’). We used adenine or guanine (A/G) for pairing with the thymine glycol residue. Afterwards, 1μg of TcNTH1 native recombinant protein was incubated with 2 pg of the oligo substrate in Endonuclease III (Endo III) buffer solution (New England Biolabs, 20 mM Tris-HCl pH 8.0, 1 mM EDTA, 1 mM DTT) for 1 h at 37°C. To inactivate the enzyme, samples were heated at 75°C for 10 min and one volume of formamide loading buffer (96% v/v formamide, 20 mM EDTA, 5 mM Tris pH 7.5, xylene cyanol 0.05% p/v, bromophenol blue 0.05% p/v) was added. After heating at 95°C for 5 min the samples were electrophoretically separated in denaturing 6M Urea-20% acrylamide gels. Labeled oligos were detected using a phosphorimager device (BioRad). As a negative control the untreated oligo was used. As positive control, the oligo was incubated with 1U Endo III (*E*. *coli* NTH1, New England Biolabs) in an Endo III buffer solution. A bifunctional DNA glycosylase activity should generate a radioactive labeled 17 mer fragment.

The monofunctional DNA glycosylase activity of the purified recombinant TcNTH1 from *E*. *coli* and from *T*. *cruzi* epimastigotes was also determined using the same oligo substrate as above. For this purpose, recombinant TcNTH1 protein was coincubated with 100 ng of purified recombinant TcAP1 *T*. *cruzi* AP endonuclease (previously generated in our lab, [23]). A monofunctional TcNTH1 DNA glycosylase activity should generate an AP site without DNA cleavage. This AP site is substrate for TcAP1 DNA cleavage that generates a radioactive labeled 17 mer fragment.

### 2.8 AP endonuclease activity assay

To determine the TcNTH1 AP endonuclease activity a 25 mer synthetic DNA oligonucleotide with a uracil at position 8 (5’-CCGCTAGUGGGTACCGAGCTCGAAT-3’) was labeled at the 5’ end with [γ-^32^P]ATP (3x10^3^Ci/mmol, 20 μCi per 40 pmol of substrate) using the DNA 5’ End Labeling System kit (Promega). This oligo was then hybridized with an unlabeled complementary oligonucleotide and incubated with 1 U of an *E*. *coli* uracil-DNA-glycosilase (New England Biolabs) to generate an AP site (oligo AP). 1 μg of purified TcNTH1 native recombinant protein (from both recombinant *E*. *coli* and recombinant epimastigotes) was incubated with 2 pg of the oligo AP in BER buffer solution (50 mM Hepes KOH pH7.8, 0.36% p/v BSA, 70 mM KCl, 5 mM MgCl2 and 0.5 mM DTT) for 1 h at 37°C. To inactivate the enzyme, samples were heated at 75°C for 10 min and one volume of formamide loading buffer (96% v/v formamide, 20 mM EDTA, 5 mM Tris pH 7.5, xylene cyanol 0.05% p/v, bromophenol blue 0.05% p/v) was added. After heating at 95°C for 5 min the samples were electrophoretically separated in denaturing 6M Urea-20% acrylamide gels. Labeled oligos were detected using a phosphorimager device (BioRad). As a negative control the untreated oligo AP was used. As positive control, the oligo AP was incubated with 1U Endo III (*E*. *coli* NTH1, New England Biolabs), or with 2U Exonuclease III (Exo III, *E*. *coli* AP endonuclease, New England Biolabs) or with 1 μg of purified recombinant TcAP1 *T*. *cruzi* AP endonuclease. An AP endonuclease activity should generate a radioactive labeled 7 mer fragment. A densitometric analysis of bands was performed using the Quantity One (Bio Rad) version 4.6.3 program.

### 2.9 *In silico* structural assessment of TcNTH1

Comparative modeling techniques were employed to enable the structural assessment of TcNTH1 and its DNA-binding properties. Structural models were generated by Modeller 9.12 [34] based on the previously published structure of *Geobacillus stearothermophilus* Endonuclease III (PDB 1P59), bound to a dideoxyribose-containing DNA molecule. The pairwise sequence alignment between TcNTH1 and 1P59 was constructed by Promals3D [35] and assessed to ensure the correct alignment of important residues. One hundred candidate structures were generated for TcNTH1 and further evaluated regarding a combination of their stereochemical properties and energy profiles, provided by Procheck [36] and ProSA [37], respectively. More details of this protocol are described on a previous article [38].

Molecular docking calculations were performed by the Haddock web server [39] on the guru interface. Active residues were defined based on the report by Fromme and Verdine [40] regarding the *G*. *stearothermophilus* protein. Passive residues were automatically defined as all residues within a 6.5 Å radius of active residues. Both active and passive residues were set for the dynamical assessment of the protein-DNA binding. The DNA molecule used for docking calculations was generated by 3D-DART [41] based on the molecule crystallized with the *G*. *stearothermophilus* Endonuclease III. All structures were visualized and assessed with Pymol [42].

### 2.10 Expression and subcellular location of TcNTH1-GFP fusion protein in transfected epimastigotes

TcNTH1-GFP fusion protein presence was detected in transfected epimastigotes homogenates by western blot assays using an anti-GFP antibody.

Subcellular location of TcNTH1-GFP was assayed by immunodetection of GFP on transfected parasites smears fixed in 70% ice methanol for 30 min. Following fixation, samples were treated with blocking solution (BSA 1% p/v, saponine 0.1%v/v, calf serum 3% v/v in PBS) for 2 h at 37°C and incubated overnight at 4°C with a monoclonal anti-GFP antibody (Thermo Scientific). Samples were then washed and incubated with a secondary antibody conjugated to Alexa 488 fluorochrome (Molecular Probes). Nuclear and kinetoplastid DNA were labeled with 4',6-diamidino-2-phenylindole (DAPI). Samples were evaluated by fluorescence microscopy observation using a 430±20 nm and a 520±20 nm filters for blue and green fluorescence, respectively. Photographs were processed computationally to determine the overlap of DAPI (pseudocolor red) and Alexa fluor 488 (green).

### 2.11 Viability assay of transfected epimastigotes exposed to H_2_O_2_

Viability of *T*. *cruzi* epimastigotes overexpressing His-TcNTH1 and exposed to H_2_O_2_ was evaluated by the AlamarBlue (Invitrogen) [43,44] and by the MTT (Sigma) [3-(4,5-dimethylthiazol-2-yl)-2,5-diphenyl tetrazolium bromide] assays. 12x10^6^ parasites/ml were incubated for 30 min with different concentrations of H_2_O_2_ (250, 500 or 1000 μM; Sigma) in LIT culture medium at 28°C. The parasites were then washed once with PBS and incubated with fresh medium for 4 h. Subsequently, parasites were resuspended in Grace’s medium and 100 μl aliquots were incubated with 10 μl of AlamarBlue reagent or with 10 μl of a MTT solution (5 mg/ml MTT plus 0.22 mg/ml phenazine metosulfate) in 96 well plates. For AlamarBlue, after additional incubation for 4 hrs at 28°C fluorescence in each well was measured at 560/590 excitation/emission (Varioskan^TM^ Flash Multimode Reader, Thermo Scientific). For the MTT assay, after an additional incubation for 4 h, the generated water insoluble formazan dye was dissolved in 100 μl of 10% w/v SDS/0.01M HCl. Optical density (OD) in each well was determined using a microplate reader (Multiskan FC, Thermo Scientific) at 570 nm. To generate sustained oxidative conditions, 20x10^6^ parasites were incubated for 24 h in the presence of 5mM glucose plus 50, 75 and 100mU glucose oxidase from *Aspergillus niger* (Sigma) at 28°C. These conditions generate a 45–65mM H_2_O_2_ concentration as measured by the Amplex Red Hydrogen/Peroxide/Peroxidase kit (Invitrogen). Parasites were then washed and their viability was evaluated by the AlamarBlue assay as described above.

### 2.12 Statistical analysis

Statistical analyses were performed using the GraphPad Prism 5.0 program. All experiments were done in triplicate and results correspond to means ± SEM from at least three independent experiments. Significant data differences were analyzed applying the two-way ANOVA with Bonferroni posttest.

## 3. Results

### 3.1 Identification and characterization of the TcNTH1 deduced protein

**Fig 1:**
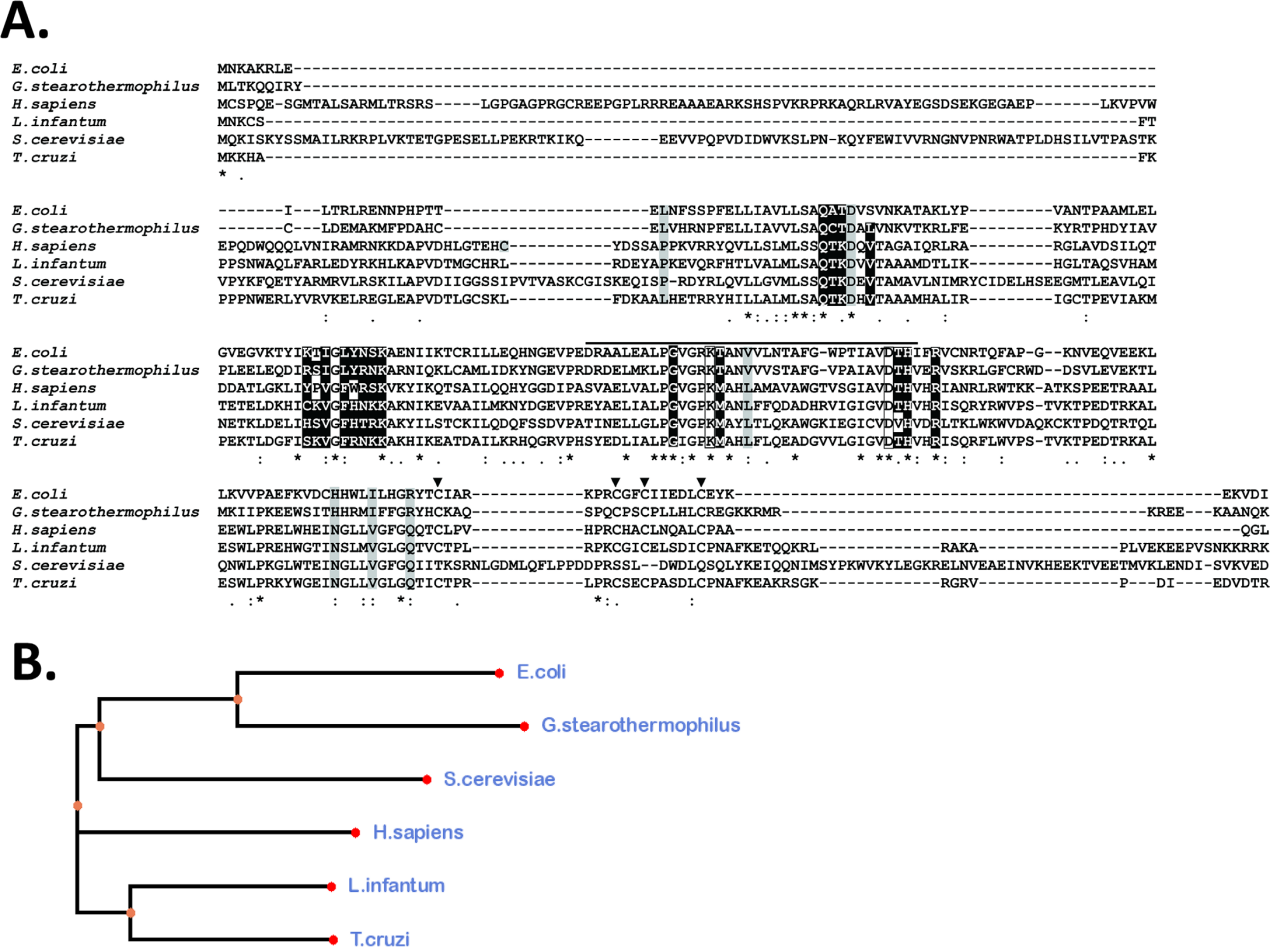
**Multiple amino acid sequences alignment (A) and deduced cladogram (B) of *T*. *cruzi* NTH1 with NTH1 from *Escherichia coli*, *Geobacillus stearothermophilus*, *Homo sapiens*, *Schizosaccharomyces cerevisiae* and *Leishmania infantum*.** (*) are identical residues match and (“:” and “.”) are chemically similar residues. Black highlighted are the residues involved in DNA recognition and binding. Gray highlighted are the residues that generates the lesion DNA base recognition pocket. Boxes are critical residues for catalysis. Arrow-heads (▼) are [4Fe-4S]^2+^ clusters union residues. Overline indicates the helix-hairpin-helix (HhH) motif.

An orthologous DNA coding sequence for a TcNTH1 DNA glycosylase protein (756 bp, GeneBank accession number 71412347) was detected in the *T*. *cruzi* genome corresponding to *Homo sapiens* NTH1 (GeneBank accession number U79718) and *E*. *coli* Endo III (GeneBank accession number AIFA01000047, locus tag ECDEC2A_2057). This gene was amplified from genomic *T*. *cruzi* DNA by PCR and inserted in a pGEM-T easy plasmid (data not shown). The deduced amino acid sequence codes for 251 residues with a predicted protein of 28.1 kDa and an isoelectric point of 9.51. Fig 1A shows a multiple deduced amino acid sequences alignment of *T*. *cruzi* TcNTH1 with *E*. *coli* Endo III, the crystallized Endo III from *Geobacillus stearothermophilus* (GeneBank accession number 1P59_A; [40]), *Homo sapiens* NTH1, *Schizosaccharomyces cerevisiae* Ntg1p (GeneBank accession number AJO92518.1) and *Leishmania infantum* Endo III (GeneBank accession number XP_001463464.1). TcNTH1 amino acid identity was 31.6% with *E*. *coli* Endo III, 28.5% with *G*. *stearothermophilus* Endo III, 47.5% with *H*. *sapiens* NTH1, 38.8% with *S*. *cerevisiae* Ntg1p and 59.3% with *L*. *infantum* NTH1.

Key catalytic amino acids specific of all DNA glycosylases [40,45] and cysteines that bind (4Fe-4S)^2+^ [40] present in *E*. *coli*, *G*. *stearothermophilus*, *H*. *sapiens* and *L*. *infantum* are fully conserved in the amino acid sequences of the enzymes depicted in Fig 1A, including TcNTH1 (boxed and arrowheads, respectively). Additionally, specific DNA glycosylases residues known to be located in the pocket that detects oxidative bases (in grey, [40]), residues that participate in DNA recognition and binding (in black, [40]) and residues involved in the helix-loop-helix motif (upper lined, [40,45,46]) are partially conserved (Fig 1). Therefore, TcNTH1 present sequence features that are expected for a canonic DNA glycosylase. A cladogram constructed considering the derived amino acid sequences of all NTH1 enzymes depicted in Fig 1A shows a close evolutive relationship between TcNTH1 and *L*. *infantum* NTH1 (Fig 1B).

### 3.2 TcNTH1 is expressed in the three *T*. *cruzi* cellular forms

**Fig 2:**
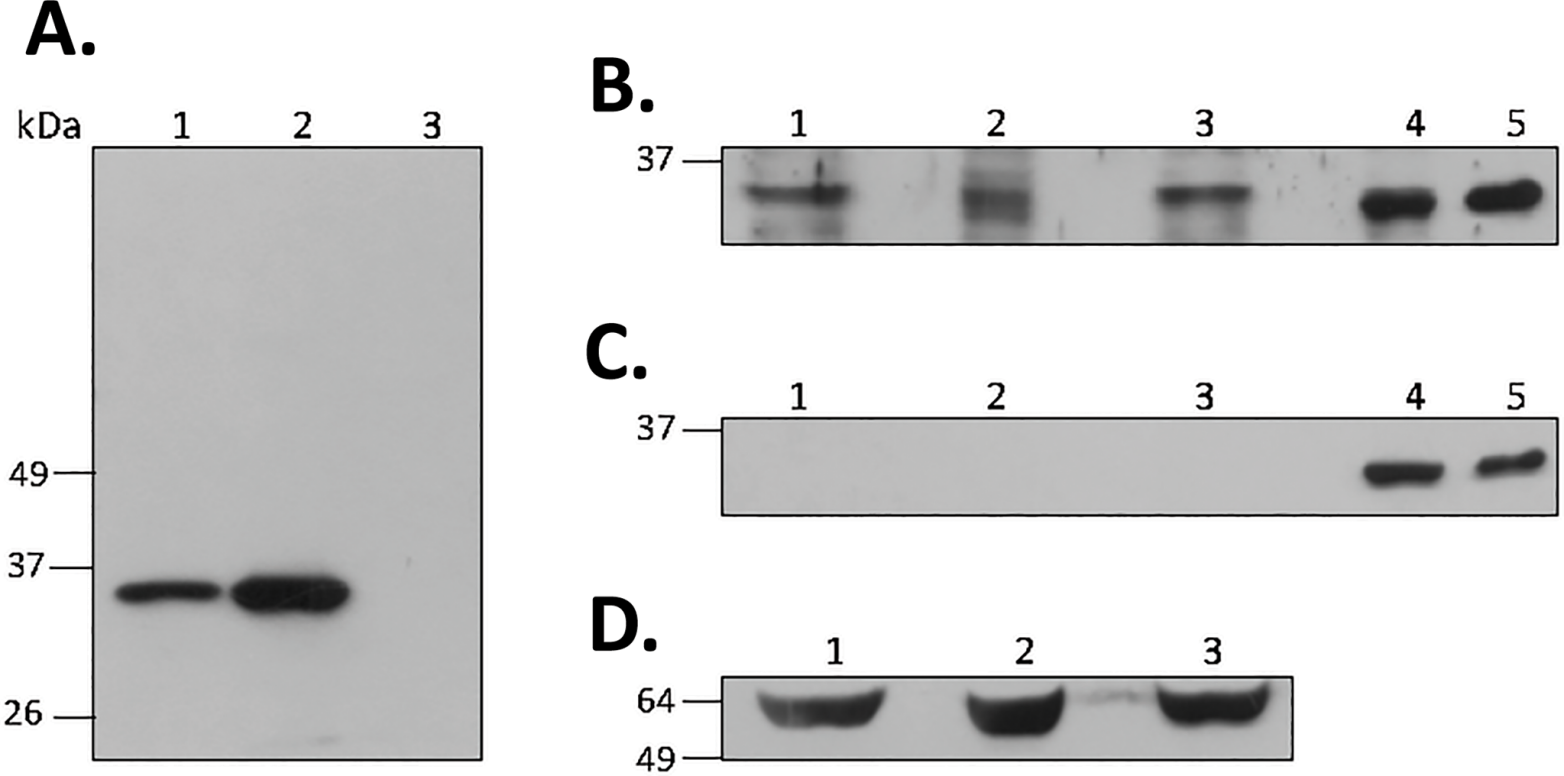
Expression of TcNTH1 DNA glycosylase in *T*. *cruzi* cellular forms. **A:** TcNTH1 polyclonal antibodies prepared in mice specifically identifies a purified recombinant TcNTH1 (lane 1) and a TcNTH1 expressed in recombinant bacterial homogenates (lane 2). This protein was not recognized in non-expressing bacteria (lane 3). **B:** Western blot detection of TcNTH1 in total protein homogenates from epimastigotes (lane 1), trypomastigotes (lane 2) and amastigotes (lane 3). Lanes 4 and 5 correspond to TcNTH1 purified from transformed *E*. *coli* and to the same protein from recombinant over-expressing *T*. *cruzi* epimastigote homogenate, respectively. **C:** Same as B but using an anti-HIS antibody. **D:** Loading control for epimastigotes (lane 1), trypomastigotes (lane 2) and amastigotes (lane 3) using an alpha-tubulin antibody. All electrophoretic separations were performed in 12%SDS-PAGE.

The recombinant TcNTH1 protein was expressed in bacteria and purified under denaturing conditions. Amino acid sequencing of this protein corresponds to a correct expression of the recombinant protein in *E*. *coli* as analyzed by MALDI-TOF (data not shown). Polyclonal antibodies prepared in mice with that recombinant protein specifically identified the purified recombinant TcNTH1 (Fig 2A, lane 1) as well as a protein of similar mass in homogenates of TcNTH1 expressing bacteria (Fig 2A, lane 2); this protein was absent in homogenates of non-expressing bacteria (Fig 2A, lane 3) and in expressing bacteria probed with pre-immune serum (data not shown). These results corroborate the antibody specificity.

Using previously described anti-TcNTH1 polyclonal antibodies, a protein of similar mass to TcNTH1 purified from transformed bacteria (Fig 2B, lane 4) or the recombinant TcNTH1 overexpressed in transfected *T*. *cruzi* epimastigote (Fig 2B, lane 5) was identified in homogenates from the two replicative parasite forms (epimastigotes, Fig 2B, lane 1 and amastigotes, Fig 2B, lane 3) as well as in the non-replicative, infective trypomastigotes (Fig 2B, lane 2). The antiHIS antibody did not detect TcNTH1 in parasite cell homogenates (Fig 2C, lanes 1, 2 and 3) but recognized the recombinant TcNTH1 purified from transformed bacteria (Fig 2C, lane 4) or the recombinant TcNTH1 overexpressed in transfected *T*. *cruzi* epimastigote (Fig 2C, lane 5). In Fig 2D, lanes 1, 2 and 3, an alpha-tubulin protein from *T*. *cruzi* epimastigote, trypomastigote and amastigote homogenates were immunodetected as loading control.

### 3.3 TcNTH1 does not present mono nor bifunctional DNA glycosylase activities but an AP endonuclease activity

**Fig 3:**
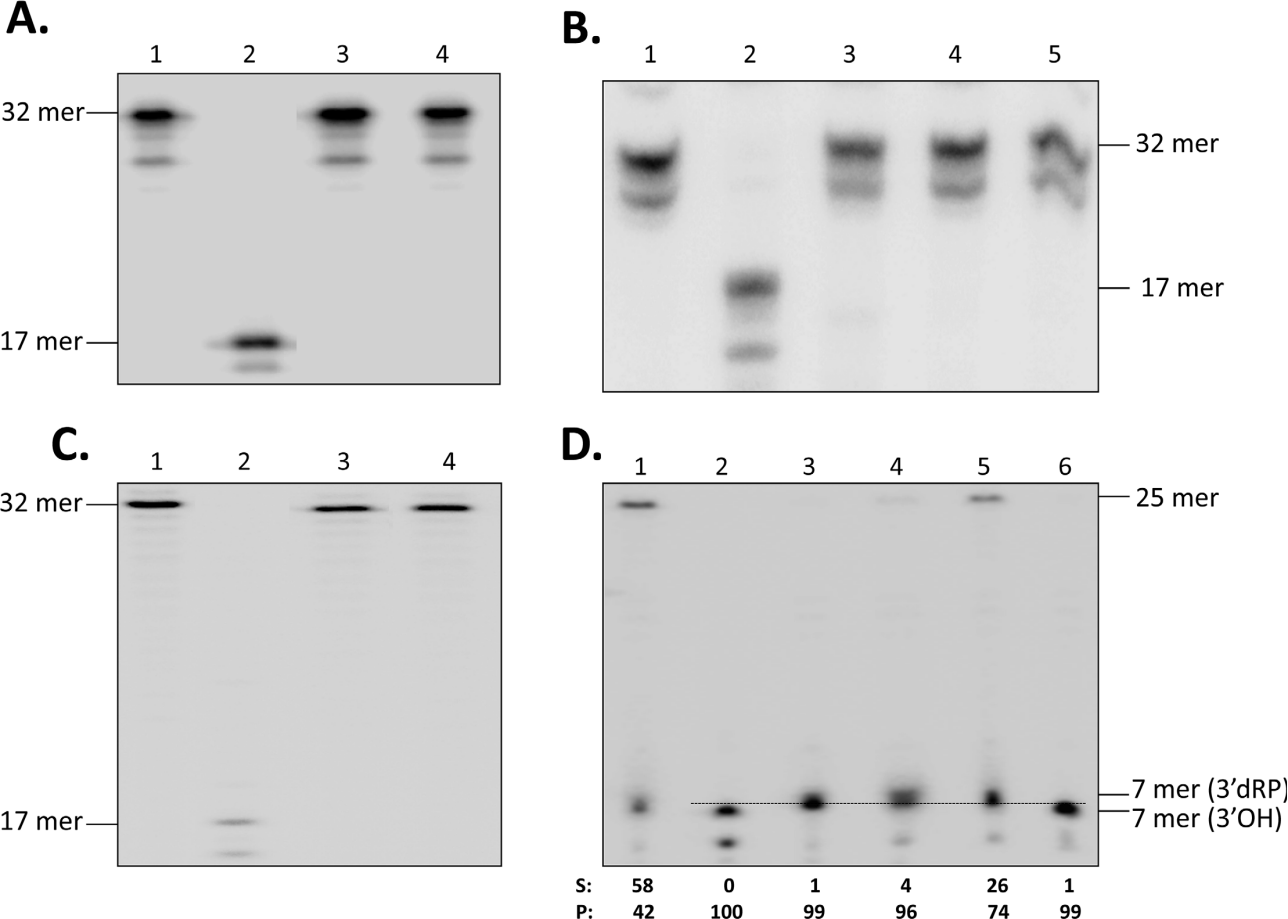
TcNTH1 does not present mono nor bifunctional DNA glycosylase activities but an AP endonuclease activity. **A, B and C:** A [γ-32P]ATP labeled 32 mer oligonucleotide containing a thymine glycol residue at position 18 incubated without enzyme (negative control, lane 1) or with *E*. *coli* Endo III (bacterial NTH1, positive control, lane 2). **A:** Lanes 3 and 4, same oligo incubated with native TcNTH1 purified from transformed bacteria or purified from transfected epimastigotes, respectively. **B:** Lane 3 same oligo co-incubated with native TcNTH1 purified from transformed bacteria and with native TcAP1 endonuclease. Lanes 4 and 5 same oligo incubated with native TcNTH1 purified from transformed bacteria or incubated with native TcAP1, respectively. **C:** Lanes 3 and 4 same oligo incubated with epimastigote or trypomastigote homogenates, respectively. **D:** A [γ-^32^P]ATP labeled 25-mer oligonucleotide with an AP site at position 8, was incubated with *E*. *coli* Endo III (AP lyase, positive control, lane 3), with native TcNTH1 purified from transformed bacteria (lane 4) and with native TcNTH1 purified from transfected epimastigotes (lane 5). Lane 1 same oligo incubated without enzyme (negative control). Lanes 2 and 6 same oligo incubated with *E*. *coli* Exo III (canonic AP endonuclease, positive control) or with TcAP1 AP endonuclease, respectively. A densitometric analysis of bands was performed using the Quantity One version 4.6.3 program (Bio Rad). S: substrate, P: product.

A TcNTH1 DNA glycosylase activity was assayed in the native recombinant TcNTH1 purified from transformed bacteria or purified from transfected epimastigotes using the classic substrate [47–49] for that enzyme (an oligonucleotide with a thymine glycol in its sequence). In spite that the gene coding for this enzyme is present in the parasite genome, that its deduced amino acid sequence presents all features expected for a canonic DNA glycosylase (Fig 1) and that it is expressed in all *T*. *cruzi* cellular forms (Fig 2B), no thymine glycol bifunctional DNA glycosylase activity was found in the parasite recombinant purified enzymes (Fig 3A, lanes 3 and 4). Negative control oligonucleotide substrate alone (Fig 3A, lane 1) is not cleaved while positive control oligonucleotide substrate incubated with *E*. *coli* Endo III (Fig 3, lane 2) produced the expected oligonucleotide cleavage. Same negative results were obtained when using the following buffers: A. 40 mM HEPES-KOH pH 7.8, 70 mM KCl, 5 mM MgCl_2_, 0.5 mM DTT, 2 mM ATP, 0.36 μg/μl BSA or B. 20 mM HEPES pH 8.0, 75 mM NaCl, 1 mM EDTA, 1 mM DTT, 100 μg/ml BSA, nor when using formamide or urea as denaturing gel conditions or when extending the incubation time from 30 to 60 min.

Those results were unexpected considering that most NTH1 orthologous DNA glycosylases are bifunctional [19,50]. To assay whether TcNTH1 is a monofunctional DNA glycosylase the same thymine glycol labeled oligonucleotide used above was co-incubated with native purified recombinant TcNTH1 enzyme and with a purified recombinant *T*. *cruzi* AP endonuclease (TcAP1), previously obtained in our laboratory [23]. The absence of a 17 mer product (Fig 3B, lane 3) confirms that TcNTH1 is not a monofunctional DNA glycosylase enzyme either. Fig 3B, lanes 1 and 2 are the negative and positive controls, respectively. Fig 3B, lane 4 shows the oligonucleotide substrate incubated with native recombinant TcNTH1 purified from bacteria. Fig 3B, lane 5 is the same substrate incubated with native recombinant TcAP1 purified from transfected epimastigotes.

Finally, using the same substrate as above the DNA glycosylase activity was assayed in epimastigote (Fig 3C, lane 3) and trypomastigote (Fig 3C, lane 4) homogenates. The absence of oligonucleotide cleavage strongly suggests that *T*. *cruzi* does not process thymine glycol in DNA by the BER pathway. Fig 3C, lanes 1 and 2 are the negative and positive controls, respectively.

### 3.4 TcNTH1 shows an AP endonuclease/lyase activity

Considering the absence of a canonic TcNTH1 DNA glycosylase activity we investigated whether this enzyme presented an AP endonuclease activity, specifically an AP lyase activity as previously reported for NTH1 in other organisms [51]. For that purpose a 5´ end-labeled 25-mer oligonucleotide with an AP site was incubated with the recombinant TcNTH1 purified under native conditions from both transformed bacteria and transfected epimastigotes. Fig 3D confirms that the activity of an *E*. *coli* Endo III (AP lyase positive control, lane 3) generates an oligo product with a slightly lower mobility than the band generated by the *E*. *coli* Exo III (control, canonic AP endonuclease, lane 2). *E*. *coli* Endo III (bacterial NTH1 orthologous) was reported to generate an oligo product with a 3' α,β-unsaturated aldehyde terminus [52,53]. That product presents a lower electrophoretic mobility than the canonic AP endonuclease product (3’OH termini) which is considered to be generated by an AP lyase activity. The similarity of the electrophoretic mobilities of bands generated in lanes 3 (positive AP lyase control), 4 (recombinant TcNTH1 purified from bacteria) and 5 (recombinant TcNTH1 purified from epimastigotes) strongly suggest that the TcNTH1 enzyme presents an AP lyase activity, as reported for orthologous enzymes in other organisms [30,53,54]. On the other hand as previously mentioned, AP endonuclease enzymes (*E*. *coli* Exo III, Fig 3D, lane 2 and recombinant *T*. *cruzi* TcAP1, lane 6) generate the expected 7 mer product proper of a canonic AP endonuclease activity (a 3′‐hydroxyl termini). In Fig 3D, lane 1, a 25 mer oligonucleotide non treated substrate (negative control) is shown; though about 50% of the AP oligo substrate is uncleaved, a 7 mer band, reported as product of AP oligo lability [55] is observed. The densitometric analysis of bands demonstrates differences between lanes 1 and 5 in Fig 3D. Clearly, recombinant TcNTH1 AP endonuclease obtained under native conditions from transfected epimastigotes presents enzymatic activity (enzyme cuts ±80% of substrate). In addition, the native recombinant TcNTH1 enzyme obtained from bacteria, cuts 96% of the substrate (Fig 3, lane 4), confirming the AP endonuclease activity of the TcNTH1 enzyme.

### 3.5 *In silico* characterization of TcNTH1 reveals an unusual DNA-binding pattern

**Fig 4:**
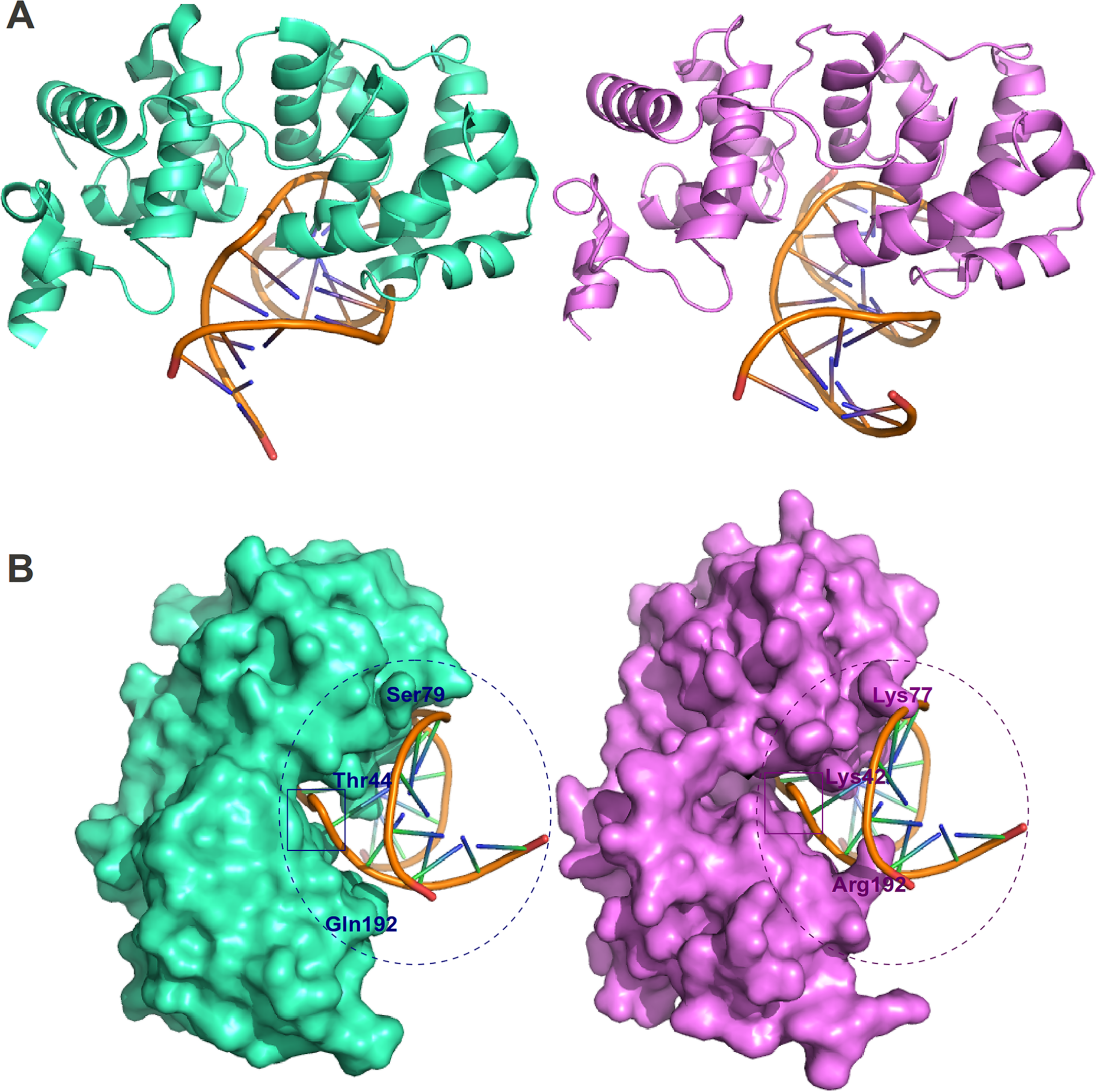
The catalytic residues disposition and its effects on lesion recognition by TcNTH1. **A:** Protein-DNA complexes of *G*. *stearothermophilus* Endonuclease III (PDB 1P59, cyan) as determined by X-ray crystallography and TcNTH1 from *T*. *cruzi* (magenta) as determined by molecular docking. Only protein backbones are shown. **B:** The same protein-DNA complexes in a different view and depicting the protein surface. Structurally divergent residues are labeled to suggest regions of interest for further analyses. The DNA-interacting region is circulated and the lesion site is shown inside the rectangle. The DNA molecule represented is from 1P59, which is in a similar position when compared to the TcNTH1-DNA complex, as shown in A.

According to BLAST results [56], the chosen structural template, 1P59, presents the higher query coverage among candidates, 81%, an e-value of 1e-21 and a similarity percentage of nearly 50% when compared with TcNTH1. The best ranked candidate structure presents 94.6% of its residues on the most favored region (the best reported percentage) of the Ramachandran plot and a ProSA z-score of -8.38 (on the top 10 best rated structures), the combination of these results representing the best quality for a candidate. Haddock has clustered 170 structures in 12 structural clusters, being the best ranked cluster also the one in which structures more closely resemble the native-like 1P59 protein-DNA complex structure. The electrostatic energy of the chosen TcNTH1-DNA complex is of -668.7 on average. The protein-DNA binding configuration for the TcNTH1 complex generated *in silico* closely resembles the crystallized *G*. *stearothermophilus* Endonuclease III (Fig 4A). Analyses of both catalytic sites suggest important differences in the position of amino acids, which could account for the lack of TcNTH1 DNA glycosylase activity on thymine glycol substrates (Fig 4B). The presence of residues within TcNTH1 lesion recognition center may impair its interaction with the damaged base, differently from *G*. *stearothermophilus* Endonuclease III.

### 3.6 A TcNTH1-GFP fusion protein expressed in *T*. *cruzi* epimastigotes is located in the parasite nuclei

**Fig 5:**
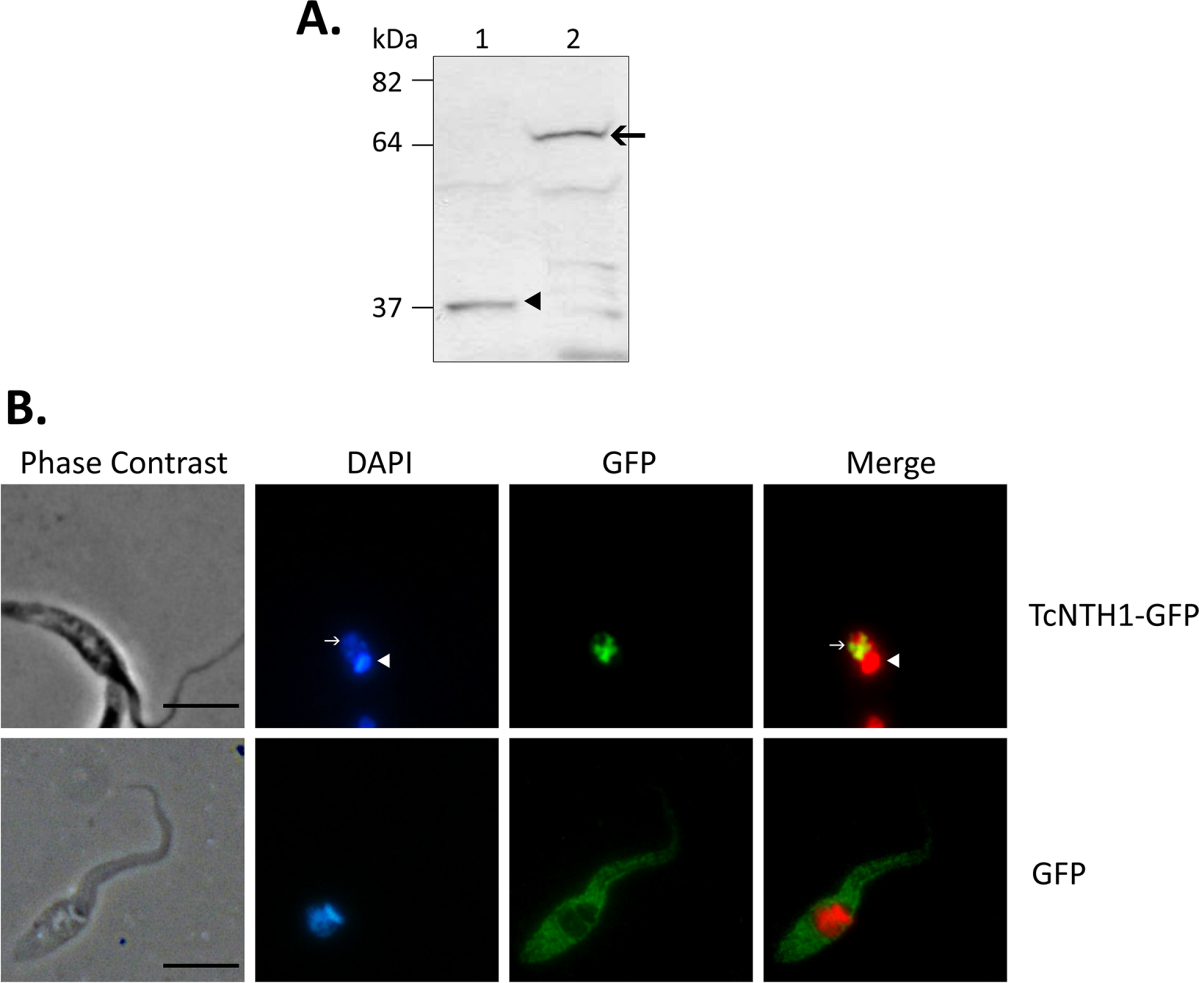
TcNTH1-GFP fusion protein is located in *T*. *cruzi* epimastigote nucleus. **A:** TcNTH1-GFP fusion protein was detected in homogenates from transfected epimastigotes by western blot assay using an anti-GFP antibody. Lane 1, GFP control protein, arrowhead. Lane 2, TcNTH1-GFP fusion protein, arrow. **B:** TcNTH1-GFP (upper row) and control GFP (lower row) proteins were detected in fixed transfected *T*. *cruzi* epimastigotes using an anti-GFP antibody and an anti-mouse secondary antibody conjugated to Alexa 488. DAPI was used for DNA detection. For merge DAPI was applied in red pseudo color. Arrows: nucleus; Arrowheads: kinetoplast. Bars 10 μm.

A TcNTH1-GFP fusion protein overexpressed in transfected epimastigotes was detected by western blot assay in parasite homogenates using an anti-GFP antibody (Fig 5A, lane 2, arrow). GFP was detected in control epimastigotes transfected with empty vector (Fig 5A, lane 1, arrowhead). TcNTH1-GFP protein was found in the parasite nucleus (Fig 5B, upper row). DAPI/TcNTH1-GFP merge demonstrate an absence of this enzyme in the *T*. *cruzi* kinetoplast (Fig 5B, upper row). Control epimastigotes transfected with empty vector show GFP evenly distributed in cytoplasm granules (Fig 5B, lower row). TcNTH1 remains in the parasite nucleus even after incubation with oxidative agents (data not shown).

### 3.7 TcNTH1 overexpression decreases parasite survival when submitted to sustained oxidative stress

**Fig 6:**
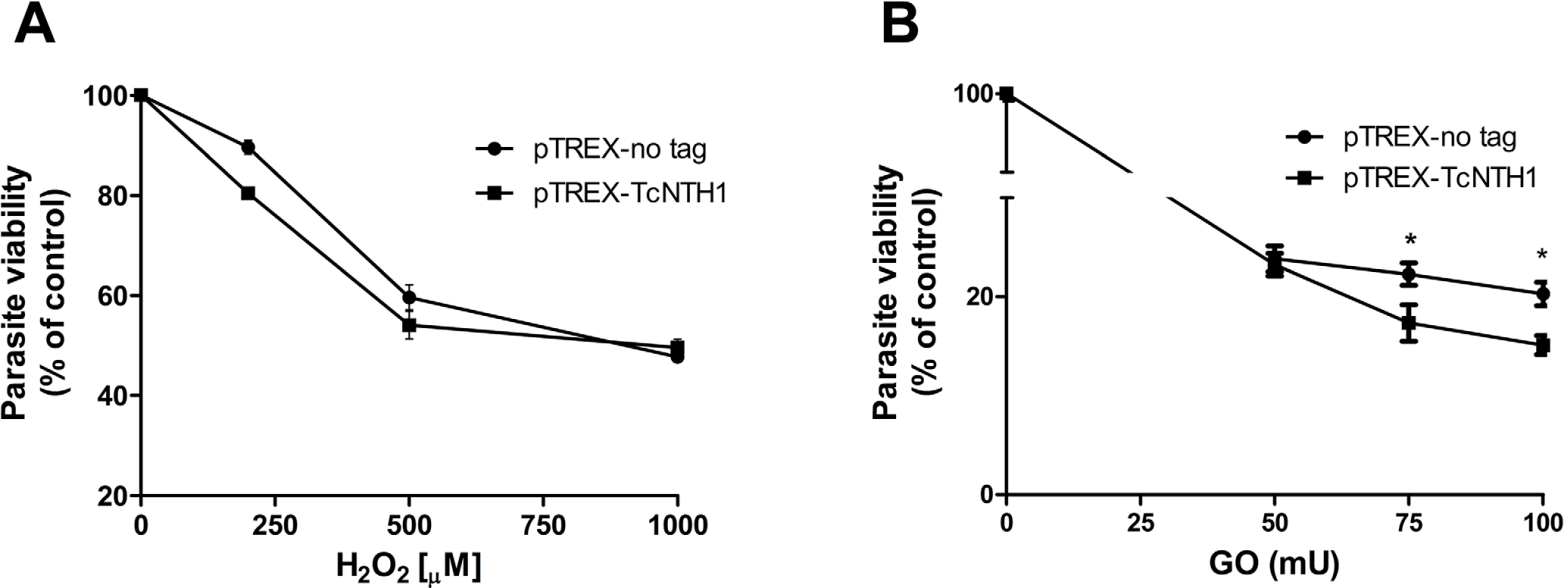
Viability of TcNTH1 transfected epimastigotes submitted to oxidative stress. TcNTH1 overexpressing epimastigotes and its control (parasites transfected with empty vector) were treated for 30 min with different H_2_O_2_ initial concentrations (A) or with a glucose-glucose oxidase system producing sustained H_2_O_2_ concentrations for 24 hours (B). Viability was determined by AlamarBlue assays. *p value: 0,01

Transfected epimastigotes overexpressing TcNTH1 maintained in the exponential phase of growth were treated for 30 min with increasing H_2_O_2_ concentrations. Afterwards parasites were incubated for 4h in fresh SFB supplemented medium to allow DNA repair [21] and their viability was measured by the AlamarBlue (Fig 6A) and the MTT (data not shown) assays. Overexpression of the TcNTH1 enzyme does not modify epimastigote viability when parasites are submitted to acute oxidative stress. On the contrary, parasite survival is decreased when TcNTH1 transfected *T*. *cruzi* epimatigotes are submitted to a sustained production of H_2_O_2_ (Fig 6B).

## 4. Discussion

*T*. *cruzi* is a flagellate protozoan from the Kinetoplastida order and Trypanosomatidae family. The Kinetoplastida order is proposed to be one of the oldest in the eukaryote phylogenetic tree, presenting an unusual expanded mitochondrium containing 15 to 30% of the cellular DNA and different cellular forms through their life cycle [57,58]. Trypanosomatids are further characterized by presenting other unique biological features, among others absence of chromatin condensation during mitosis, polycistronic mRNA synthesis, nuclear trans-splicing and mitochondrial mRNA editing [59–63]. *T*. *cruzi* is a diploid organism [64,65] and according to genomic analysis and synteny maps the number of chromosomes varies across different strains [66], but is stable during the parasite cell cycle [67].

Although all cellular forms of *T*. *cruzi* are exposed to both oxygen and nitrogen reactive species the parasite is able to survive in its hosts [11,13,14,16,68] in spite that the hostile environment induces DNA damage [21]. From the many mechanisms of DNA repair the BER pathway is known to be present and active in *T*. *cruzi* [18,21,23,69]. In this pathway there are several steps that are initiated by DNA glycosylases, followed by AP endonucleases, DNA polymerases and DNA ligases [19,27]. Some DNA glycosylases and two AP endonucleases were previously described in *T*. *cruzi* [22,23,70,71]. We hereby report the presence and activity of a NTH1 DNA glycosylase in *T*. *cruzi* (TcNTH1). In other eukaryoyes NTH1 was found to be important for the detection and excision of oxidized pyrimidines as an initiation step of the BER pathway [49,72–74].

Endo III NTH1 DNA glycosylase was discovered, purified and characterized in *E*. *coli*, being considered as an endonucleolytic enzyme [75]. DNA sequences showing homology regions with that enzyme were found in diverse organisms from Bacteria, Archea and Eukarya [76]. In HeLa cells NTH1 was located in nucleus and cytoplasm though in other cells it was mainly concentrated in the nucleus [51,72]. In Trypanosomatid sequences encoding for Endonuclease III orthologous were described in *Leishmania infantum*, *Leishmania major*, *Trypanosoma brucei* and *T*. *cruzi* [77].

Using a specific polyclonal antibody a TcNTH1 was identified in the three cellular forms of *T*. *cruzi*, showing a molecular mass of approximately 37 kDa, which is in the range of NTH1 from other organisms [49,51,78–83]. This result suggests that the enzyme is constitutively expressed, its presence non-depending upon the proliferative or non-proliferative or differentiate stages of the parasite. Considering the permanent exposure to oxidative species generated by the parasite itself or as a defense mechanism by its hosts the constitutive character of this enzyme is also in accordance with its proposed role in DNA repair [11,13,16,21,23,84].

Though *E*. *coli* NTH1 was considered an endonuclease [75], it works by a ß-elimination mechanism generating a product that is not recognized by a DNA polymerase [53]. At present, *E*. *coli* NTH1 is described as a bifunctional enzyme presenting both DNA glycosylase and AP lyase activities, recognizing and eliminating a wide variety of pyrymidine oxidative derivates and generating a 3’α,ß-non-saturated aldehide [47,53,85–88].

The enzymatic activity of TcNTH1 was assayed using a [γ-^32^P]ATP labeled oligo with a thymine glycol (Tg) paired to either adenine or guanine. If TcNTH1 is a bifunctional enzyme it should eliminate Tg and cut the oligo used as substrate. However, TcNTH1 purified from transformed bacteria as well as TcNTH1 purified from transfected epimastigotes did not cut the Tg oligo (see Fig 3A). This result is unexpected considering that NTH1 from other organisms such as *S*. *pombe*, *C*. *elegans*, *M*. *musculus*, and *H*. *sapiens* present catalytic activity on oligonucleotides with a Tg [29,49,51,79,83,89]. Moreover, human as well as *C*. *elegans* NTH1 present high catalytic specificity on Tg oligonucleotides, as the one used in our studies [48,49].

To assay whether TcNTH1 is a monofunctional DNA glycosylase the same Tg labeled oligonucleotide used above was co-incubated with a native purified recombinant TcNTH1 enzyme and a native purified recombinant *T*. *cruzi* AP endonuclease (TcAP1), previously obtained in our laboratory [23]. Results confirm that, under those experimental conditions, TcNTH1 is not a monofunctional DNA glycosylase enzyme either. In our knowledge, *T*. *cruzi* is the first organism showing a NTH1 orthologous that does not processes a thymine glycol substrate. Moreover parasite homogenates were not able to process the Tg oligonucleotide either. These unexpected results suggest that in *T*. *cruzi* the BER pathway is not involved in the thymine glycol elimination leading to DNA repair. However, we cannot discard that DNA base lesions other than Tg may be processed by TcNTH1.

Along evolution the oxidation of thymine to thymine glycol upon oxidative DNA damage is almost universal and the presence of this modified base in DNA impairs replication [90] and in some cases transcription [91,92]. Therefore, this oxidative DNA lesion should be repaired. As the *T*. *cruzi* DNA repair BER pathway does not present the capacity of repairing those lesions, our results point to some other mechanism for Tg elimination in *T*. *cruzi*, such as the NER (nucleotide excision repair) pathway, as proposed for other organisms [92]. However, we cannot evaluate whether the NER pathway processes Tg in DNA by incubation of our substrate with *T*. *cruzi* homogenates considering that a much longer substrate is needed for the NER DNA repair mechanism to proceed [93,94].

The fact that TcNTH1 does not eliminate thymine glycol from DNA cannot be explained by the absence or alteration of amino acids from sites required for its catalytic function, since amino acid residues that participate both in the DNA binding and catalysis are reasonably well conserved (See Fig 1). Based on the *in silico*-generated TcNTH1 protein-DNA complex we have investigated the DNA-binding properties of the *T*. *cruzi* protein. As a striking feature, we observed the position of some TcNTH1 residues to be significantly divergent from their counterparts in the 1P59 structure, increasing the active site occupancy and likely precluding the correct positioning of thymine glycol (Fig 4). This would suggest a better recognition of the DNA lesion by the *G*. *stearothermophilus* protein in comparison to TcNTH1, thus corroborating the observed absence of a thymine glycol removing function.

Interestingly, TcNTH1 presented an AP endonuclease activity, specifically an AP lyase activity as previously reported in other organisms [29,49,51]. The native recombinant TcNTH1 enzymes purified from bacteria or from the parasite were incubated with a 25 mer substrate presenting an apurinic-apyrimidinic site. Interestingly, this substrate was cut by both enzymes as well as by *E*. *coli* Exo III, *E*. *coli* Endo III and the *T*. *cruzi* endonuclease TcAP1, used as controls. However, while Exo III and TcAP1 produced the expected 7 mer oligo, the product obtained with the recombinant TcNTH1 purified from bacteria or recombinant TcNTH1 purified from *T*. *cruzi* presented a lower electrophoretic mobility that is similar to the product obtained with *E*. *coli* Endo III (see Fig 3D). Considering that *E*. *coli* Endo III is a bifunctional enzyme that functions by ß-elimination [85] while Exo III is an endonuclease with hydrolytic action on the phosphodiester linkage [95], our results suggest that TcNTH1 is an AP endonuclease presenting an AP lyase activity.

All along evolution AP lyase activity of the BER pathway is observed in the nucleus mainly as a result of DNA-polymerase beta activity. Contrarily, in *T*. *cruzi* two beta-polymerases were described, both as mitochondrial enzymes [96]. Taking this into account, TcNTH1 may be responsible of AP lyase activity in the *T*. *cruzi* nuclei. This is in agreement with the nuclear location of the TcNTH1 in this parasite.

The fact that transfected parasites over-expressing TcNTH1 do not modify their viability when exposed for a short time to H_2_O_2_, as compared with parasites transfected with empty vector, suggests that the endogenous enzyme is sufficient to maintain DNA integrity under those conditions. However, a sustained exposure of TcNTH1 over-expressing parasites to H_2_O_2_ decreases their viability. A similar result was observed when TcOGG1 overexpressing parasites were exposed to H_2_O_2_ for extended periods [22]. These results may be explained considering that an overexpression of DNA glycosylases in different eukaryotic cells may induce AP sites and strand breaks in DNA [97,98] in a time depending manner, such as a glucose-glucose oxidase incubation for 24h, thus decreasing parasite viability.

Our results point to the expression of an active NTH1 DNA glycosylase in *T*. *cruzi* that shows enzymatic features different from those reported for recent eukaryotes. Accordingly, other DNA repair mechanism should be expected leading to eliminate thymine glycol from oxidized parasite DNA. Furthermore, TcNTH1 may play a role in the AP site recognition and processing.
